# Supplementary material for: Metabolomic analysis of Drosophila melanogaster larvae lacking Pyruvate kinase
Source: bioRxiv. 2023 Jun 19:2023.06.05.543743. Originally published 2023 Jun 7. Preprint. [Version 2] doi: 10.1101/2023.06.05.543743 (PMC10274742; doi:10.1101/2023.06.05.543743)
Supplement: Supplement 1 — Figure S1. Pyk mRNA transcript levels are significantly reduced in Pyk mutant larvae. Total RNA from stage w1118; Pykprec control larvae and w1118; Pyk60/61 mutant larvae were analyzed by northern blot hybridization to detect transcripts encoding Pyk, CG18596, and Polr3F. Hybridization to detect rp49 mRNA is included as a loading control. [file media-1.pdf]

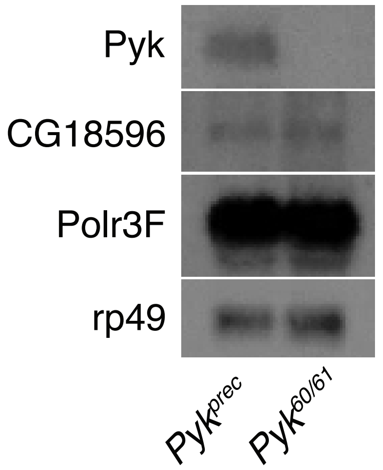

Figure S1. *Pyk* mRNA transcript levels are significantly reduced in *Pyk* mutant larvae. Total RNA from stage w^{1118}; Pyk^{prec} control larvae and w^{1118}; $Pyk^{60/61}$ mutant larvae were analyzed by northern blot hybridization to detect transcripts encoding Pyk, CG18596, and Polr3F. Hybridization to detect *rp49* mRNA is included as a loading control.
