# Supplementary material for: Metabolomic analysis of Drosophila melanogaster larvae lacking Pyruvate kinase
Source: bioRxiv. 2023 Jun 19:2023.06.05.543743. Originally published 2023 Jun 7. Preprint. [Version 2] doi: 10.1101/2023.06.05.543743 (PMC10274742; doi:10.1101/2023.06.05.543743)
Supplement: Supplement 2 — Figure S2. A comparison of sequence similarities between Pyk homologs. A heatmap of pairwise sequence identities extracted from the ensemble MSA. Species abbreviations: Aa, A. aegypti; Dm, D. melanogaster; Hs, H. sapiens; Mm, M. musculus; Ce, C. elegans; Sc, S. cerevisiae; At, A. thaliana; Ec, E. coli. See Supplemental Table 1 for a list of isoforms used in this analysis. [file media-2.pdf]

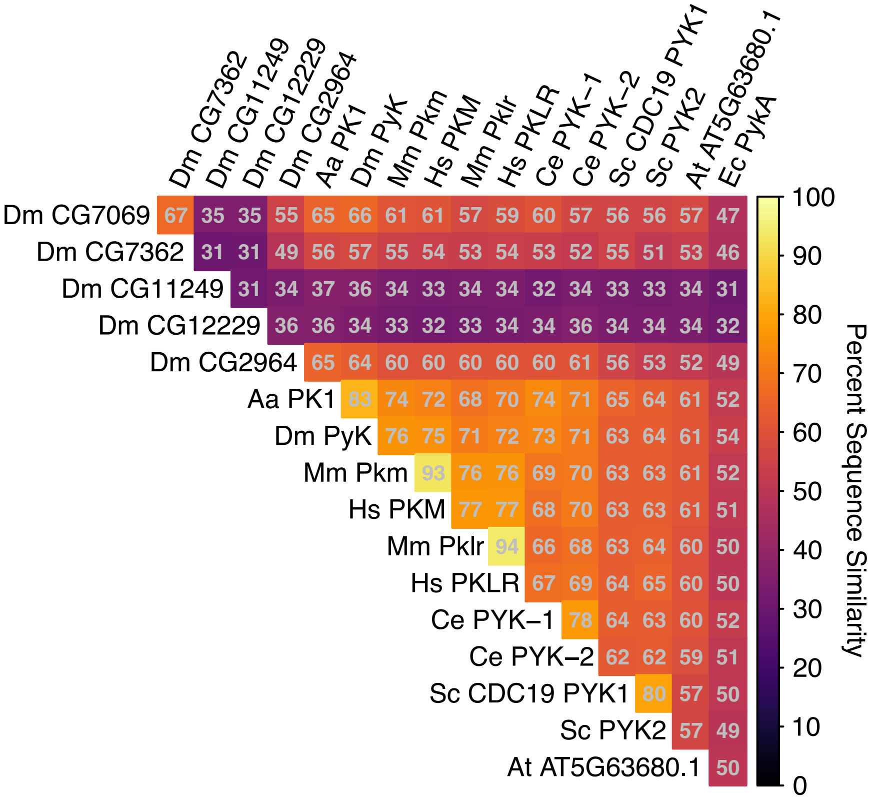

Figure S2. A comparison of sequence similarities between Pyk homologs. A heatmap of pairwise sequence identities extracted from the ensemble MSA. Species abbreviations: Aa, *A. aegypti; Dm*, *D. melanogaster*; Hs, *H. sapiens*; Mm, *M. musculus*; Ce, *C. elegans*; Sc, *S. cerevisiae*; At, *A. thaliana*; Ec, *E. coli.* See Supplemental Table 1 for a list of isoforms used in this analysis.
