# Supplementary material for: Metabolomic analysis of Drosophila melanogaster larvae lacking Pyruvate kinase
Source: bioRxiv. 2023 Jun 19:2023.06.05.543743. Originally published 2023 Jun 7. Preprint. [Version 2] doi: 10.1101/2023.06.05.543743 (PMC10274742; doi:10.1101/2023.06.05.543743)
Supplement: Supplement 3 — Figure S3. A comparison of the metabolomic data from Pyk23/31 mutant and Pyk21/+ control samples using principal component (PC) analysis. Targeted metabolomics data from Table S1 was analyzed using principal component analysis. Analysis was conducted using Metaboanalyst 5.0. [file media-3.pdf]

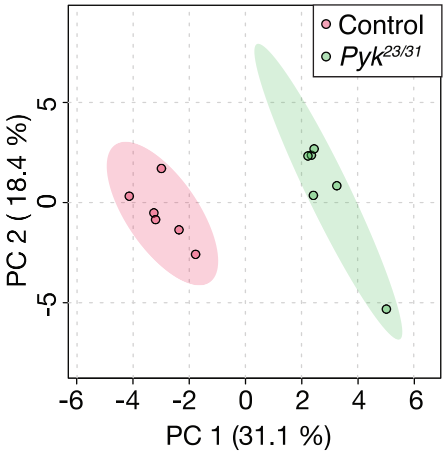

Figure S3. A comparison of the metabolomic data from *Pyk*$^{23/31}$ mutant and *Pyk*$^{21/+}$ control samples using principal component (PC) analysis. Targeted metabolomics data from Table S2 was analyzed using principal component analysis. Analysis was conducted using Metaboanalyst 5.0.
