# Supplementary material for: Metabolomic analysis of Drosophila melanogaster larvae lacking Pyruvate kinase
Source: bioRxiv. 2023 Jun 19:2023.06.05.543743. Originally published 2023 Jun 7. Preprint. [Version 2] doi: 10.1101/2023.06.05.543743 (PMC10274742; doi:10.1101/2023.06.05.543743)
Supplement: Supplement 4 — Figure S4. A comparison of the metabolomic data from Pyk60/61 mutant and Pykprec control samples using principal component (PC) analysis. Targeted metabolomics data from Table S2 was analyzed using principal component analysis. Analysis was conducted using Metaboanalyst 5.0. [file media-4.pdf]

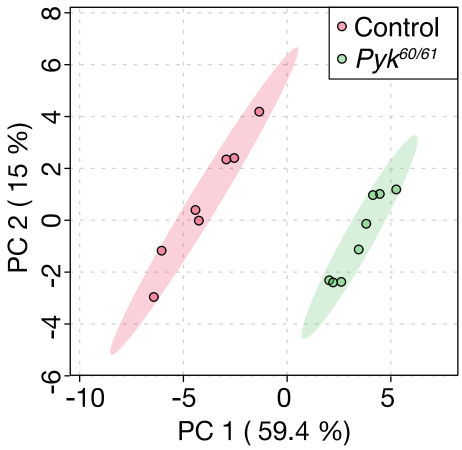

Figure S4. A comparison of the metabolomic data from *Pyk*$^{60/61}$ mutant and *Pyk*^{prec} control samples using principal component (PC) analysis. Targeted metabolomics data from Table S3 was analyzed using principal component analysis. Analysis was conducted using Metaboanalyst 5.0.
